# Sublethal heat treatment enhances lactic acid uptake in macrophages via MCT1, leading to reduced paraspeckle formation and a subsequent decrease in macrophage pyroptosis

**DOI:** 10.3389/fimmu.2023.1290185

**Published:** 2024-01-11

**Authors:** Zhuoyang Fan, Guowei Yang, Rongkui Luo, Xudong Qu, Xiaodan Ye, Jianhua Wang, Zhiping Yan, Minfeng Shu, Wei Zhang, Rong Liu

**Affiliations:** ^1^ Department of Interventional Radiology, Zhongshan Hospital, Fudan University, Shanghai, China; ^2^ Shanghai Institute of Medical Imaging, Fudan University, Shanghai, China; ^3^ National Clinical Research Center for Interventional Medicine, Zhongshan Hospital, Fudan University, Shanghai, China; ^4^ Department of Pathology, Zhongshan Hospital of Fudan University, Shanghai, China; ^5^ Department of Radiology, Zhongshan Hospital of Fudan University, Shanghai, China; ^6^ Department of Pharmacology, School of Basic Medical Sciences, Shanghai Medical College, Fudan University, Shanghai, China; ^7^ Department of Interventional Radiology, Zhongshan Hospital (Xiamen), Fudan University, Xiamen, China

**Keywords:** heat ablation, lactic acid, M2 polarization, paraspeckle, pyroptosis

## Abstract

**Introduction:**

Heat ablation is one of the key modalities in treating liver cancer, yet the residual cancer tissues suffering sublethal heat treatment possess a potential for increased malignancy. This study conducts a comprehensive analysis of cellular dynamics, metabolic shifts, and macrophage polarization within the tumor microenvironment following sublethal heat treatment.

**Methods:**

We observed significant acidification in tumor cell supernatants, attributed to increased lactic acid production. The study focused on how this pH shift, crucial in tumor progression and resistance, influences macrophage polarization, especially towards the M2 phenotype known for tumor-promoting functions. We also examined the upregulation of MCT1 expression post sublethal heat treatment and its primary role in lactic acid transport.

**Results:**

Notably, the study found minimal disparity in MCT1 expression between hepatocellular carcinoma patients and healthy liver tissues, highlighting the complexity of cancer biology. The research further revealed an intricate relationship between lactic acid, MCT1, and the inhibition of macrophage pyroptosis, offering significant insights for therapeutic strategies targeting the tumor immune environment. Post sublethal heat treatment, a reduction in paraspeckle under lactic acid exposure was observed, indicating diverse cellular impacts. Additionally, PKM2 was identified as a key molecule in this context, with decreased levels after sublethal heat treatment in the presence of lactic acid.

**Discussion:**

Collectively, these findings illuminate the intertwined mechanisms of sublethal heat treatments, metabolic alterations, and immune modulation in the tumor milieu, providing a deeper understanding of the complex interplay in cancer biology and treatment.

## 1 Introduction

Hepatocellular carcinoma (HCC) is a predominant malignancy globally, especially in Asian and African populations. It arises due to multiple factors, including viral hepatitis, chronic alcohol consumption, and non-alcoholic fatty liver disease (NAFLD) (1). Diagnosis mainly relies on radiological and serological evaluations. While heat ablation (such as microwave or radiofrequency ablation) is employed for HCC treatment, its outcomes, especially with Sublethal Heat Treatment (SLHT), are often suboptimal (2).

In the realm of tumor immunology, immunotherapy offers hope for HCC. Tumor immunity intricacies extend beyond just tumor and immune cells, involving cells like tumor-associated macrophages (TAMs) within the tumor microenvironment (3). TAMs, crucial in tumor progression, can either promote or inhibit tumor growth (4). Their role is pivotal for immunotherapeutic success, and manipulating TAM activity is challenging.

Tumor metabolism, particularly the role of lactic acid, is gaining research attention. Tumor cells, showcasing unique metabolic profiles, thrive on lactic acid, which can modify the tumor environment and weaken immune responses (5). Understanding lactic acid’s influence on tumor metabolism and immunity offers potential therapeutic insights.

Furthermore, phase separation, exemplified by paraspeckles, plays a role in tumor progression (6). With NEAT1 as their main structural component and key proteins like NONO, these structures influence various nuclear activities (7). Despite their significance, the mechanisms by which paraspeckles impact tumor immunity, especially macrophage functionality, remain unclear. Our study finds that lactic acid from SLHT promotes macrophage M2 phenotype transformation and reduces paraspeckle formation in macrophages, inhibiting their pyroptosis, and highlighting lactic acid’s crucial role in tumor immunity.

## 2 Methods

### 2.1 Cell culture and treatment

In this project, we used two human HCC cell lines, HCCLM3 and Huh7; one leukemia cell line, U937 to perform the *in vitro* study. The three cell lines were obtained from Liver Cancer Institute, Zhongshan Hospital, Fudan University (Shanghai, China). The HCC cells were cultured in Dulbecco’s modified Eagle’s medium (DMEM, Gibco, Grand Island, NY, USA), which contained 10% fetal bovine serum (FBS) and antibiotics (penicillin (100 U/ml)/streptomycin (0.1 mg/ml)). The cells were incubated at 37°C and 5% CO2 in a humidified environment, and the medium was refreshed three times a week. While U937 cells were cultured in RPMI1640 medium (Sigma-Aldrich, USA). U937 cells were differentiated into macrophages (M0φ) by exposure to phorbol 12-myristate 13-acetate (PMA) from Sigma (Saint-Quentin Fallavier, France) at a concentration of 100 ng/mL for a duration of 24 hours. To derive tumor-associated macrophages (TAM) from U937, PMA-treated U937 cells (at a density of 2 × 10^6^ cells) were seeded into the lower compartment of a transwell co-culture system. Concurrently, hepatocellular carcinoma (HCC) cells were positioned in the upper compartment, separated by a 0.4-μm porous membrane. Following a 24-hour interval, a co-culture was established between HCC cells and the U937-derived macrophages. After an additional 48 hours, the macrophages were harvested for subsequent RNA extraction and assorted assays. In our previous study, we have established the *in vitro* sublethal heat treatment (SLHT) model (8).

### 2.2 Transfection and stable cell lines

Plasmid transfection was performed using Lipofectamine 2000 Reagent (Life Technology, Thermo Fisher Scientific, DE, USA). Plasmids (pcDNA3.1-PKM2), shMCT1(shSLC16A1) virus (hU6-MCS-Ubiquitin-mCherry-IRES-Neomycin) were purchased from Genechem (Shanghai, China). An empty vector was used as a negative control. The transfection procedures were strictly followed the manufacturer’s instructions for Lipofectamine 2000 Reagent (Invitrogen). For one group, a total of 5×10^5^ cells were seeded into a well of 6-well plate. After transfection, qRT-PCR analysis or western blot analysis was used to verify the transfection efficiency.

### 2.3 RNA extraction and quantitative real-time PCR

The detailed procedure of qRT-PCR was the same as the methods described previously (9). The expression level of each gene was normalized to that of ACTIN, which served as an internal control according to the 2^−ΔΔCt^ method. Primers (synthesized by Sunya, China) for human genes and rabbit genes were as follows:

Human-ACTIN:

ACTIN-F: ACCTTCTACAATGAGCTGCG,

ACTIN-R: CCTGGATAGCAACGTACATGG.

Human-MCT1:

MCT1-F: AGGTCCAGTTGGATACACCCC,

MCT1-R: GCATAAGAGAAGCCGATGGAAAT.

Human-PKM2:

PKM2-F: ATGTCGAAGCCCCATAGTGAA,

PKM2-R: TGGGTGGTGAATCAATGTCCA.

Human-CD206:

CD206-F: TCCGGGTGCTGTTCTCCTA,

CD206-R: CCAGTCTGTTTTTGATGGCACT.

Human-CD68:

CD68-F: GGAAATGCCACGGTTCATCCA,

CD68-R: TGGGGTTCAGTACAGAGATGC.

Human-GSDMD:

GSDMD-F: GTGTGTCAACCTGTCTATCAAGG,

GSDMD-R: CATGGCATCGTAGAAGTGGAAG.

Human-CASP1:

CASP1-F: AATAAATGGCTTGCTGGATGAG,

CASP1-R: GCCGTGCTCTGCTCATCTAT.

Human-IL1β:

IL1β-F: GCCTCTAAGGACCGCAATGT,

IL1β-R: CCTCCTGGTCCTGAAGATGC.

### 2.4 Lactic acid detection

Utilizing the pH Test Strip (Ayclif, USA), we applied a minimal amount of culture medium and subsequently compared the resultant reading with the provided reference standard. The pH meter used is the Sartorius standard pH meter PB-10 (Sartorius, Germen): fully automatic temperature compensation, fully automatic display of the electrode slope and the use of the state, synchronized display of pH value. The sample supernatant is removed and immersed in the liquid to be measured with the detection end of the pH meter, and each group of samples is tested three times.

### 2.5 Western blot

Cells were harvested at the indicated times. In each pole, 20 µg of total protein was used for electrophoresis. The membrane was blocked with 5% nonfat milk at room temperature for 1 hour and then incubated with primary antibodies for overnight at 4°C. Subsequently, a corresponding secondary antibody was applied to the membrane and incubated for 2 hours at room temperature. The protein bands were visualized using a chemiluminescence ECL kit (Tanon, Shanghai, China).

The antibodies used in this study were as follows:

β-Actin (13E5) Rabbit mAb #4970(CST);

MCT1 Rabbit Polyclonal Antibody#HA500190(Huabio, China).

NONO Antibody#ab133574 (Abcam).

PKM2 (D78A4) XP^®^ Rabbit mAb #4053(CST).

Gasdermin D (E8G3F) Rabbit mAb #97558(CST).

Caspase-1 (D7F10) Rabbit mAb #3866(CST).

IL-1β (D3U3E) Rabbit mAb #12703(CST).

Anti-rabbit IgG, HRP-linked Antibody #7074(CST).

### 2.6 GEPIA and HPA and TIMER

The correlation of different expression with overall survival of HCC patient as well as HCC pathological stages was evaluated by the GEPIA2 (http://gepia2.cancer-pku.cn) and the TCGA database (http://www.oncolnc.org/). The statistical method applied was Kaplan‐Meier method with a log rank test. The expression score of HCC and normal liver tissue in the HPA database described an estimated gene level. Timer 2.0 database (http://timer.comp-genomics.org/timer/) has been widely used to explore the infiltration of immune cells around tumor. In this study, we sought the possible interactive proteins with SERPINE1.

### 2.7 ELISA

During the cultivation, the cell culture supernatant was collected. The L-Lactic Acid (LA) Colorimetric Assay Kit (#E-BC-K044-M, Elabscience Biotechnology Co.,Ltd, China) was used to analyze the lactic acid concentrations following the manufacturer’s instructions.

### 2.8 RNA scope plus immunofluorescence

The RNA scope plus IP was performed following the manufacturers’ instructions. We used RNAscope Multiplex Fluorescent ReagentKit v2(#323100), and the RNAscope Probe - Hs-NEAT1-long (#411541), which were bought from ACDBio (USA).

### 2.9 Statistical analysis

All of the analyses in this study were performed in GraphPad Prism 8.00 (GraphPad Software, San Diego, USA). Student’s *t*-test was used for statistical analysis. A *p* value less than 0.05 was considered statistically significant. Data are presented as the means ± SEM.

## 3 Results

### 3.1 After sublethal heat treatment, HCC cells exhibit an enhanced production of lactic acid

**Figure 1:**
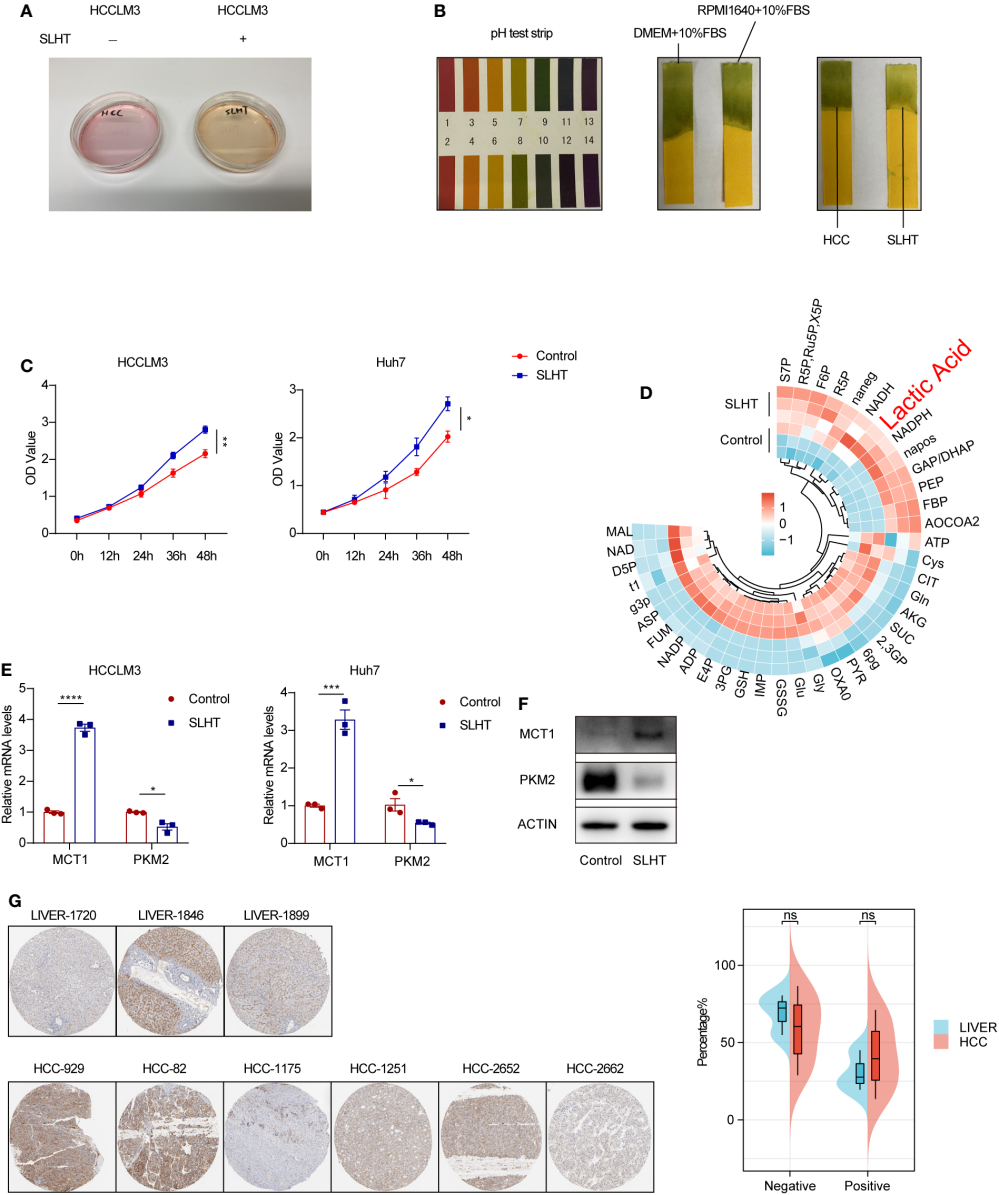
After sublethal heat treatment, HCC cells exhibit an enhanced production of lactic acid. **(A)** The culture medium of normal HCC and SLHT. **(B)** The pH test strips on different conditional medium. **(C)** ELISA of lactic acid on normal medium or SLHT medium. **(D)** Changes of LS-MS/MS of metabolites in cells of SLHT group and Control group. **(E)** qPCR of MCT1 and PKM2. **(F)** Western of MCT1 and PKM2. **(G)** Immunohistochemical pathological slide display and analysis in the HPA database. “NS”, not significant, *p < 0.05, **p < 0.01, ***p < 0.001, ****p < 0.0001.

Our observations revealed that the supernatant of tumor cells post-sublethal heat treatment presented a more yellowish hue compared to the control group (**Figure 1A**), suggesting a potential shift towards increased acidity. Subsequent analysis using pH test strips confirmed a more acidic state, with pH values approaching 7 (**Figure 1B**). We used pH meter to test the pH values, as well (**Supplementary Table S1**). While several compounds can influence pH, lactic acid has been reported to exert significant effects on tumor cells, and these cells are known to produce substantial amounts of lactic acid (10).

To delve deeper into this phenomenon, we employed an enzyme-linked immunosorbent assay (ELISA) to quantify lactic acid levels in the tumor cell supernatant at various time points post-SLHT. Our results demonstrated a marked increase in extracellular lactic acid production compared to the control group (**Figure 1C**). Moreover, utilizing mass spectrometry to assess intracellular metabolites, we noted a significant rise in lactic acid levels, which was statistically significant (**Figure 1D**). Literature indicates that MCT1 plays a crucial role as a membrane protein facilitating lactic acid transport across cellular barriers (11). Therefore, we analyzed MCT1 expression levels, and both quantitative PCR and Western blot analyses revealed an upregulated expression of MCT1 following sublethal heat treatment (**Figure 1E, F**). Interestingly, upon consulting the HPA database, we observed no significant difference in MCT1 expression between hepatocellular carcinoma patients and healthy liver tissues (**Figure 1G**). This alludes to the tumor cells’ capacity, in conjunction with their microenvironment, to regulate pH levels. In conclusion, our findings strongly suggest that the acidification observed in the tumor cell supernatant is primarily attributable to the increased production of lactic acid by the tumor cells.

### 3.2 Accelerated M2 macrophage polarization in supernatants after sublethal heat treatment

**Figure 2:**
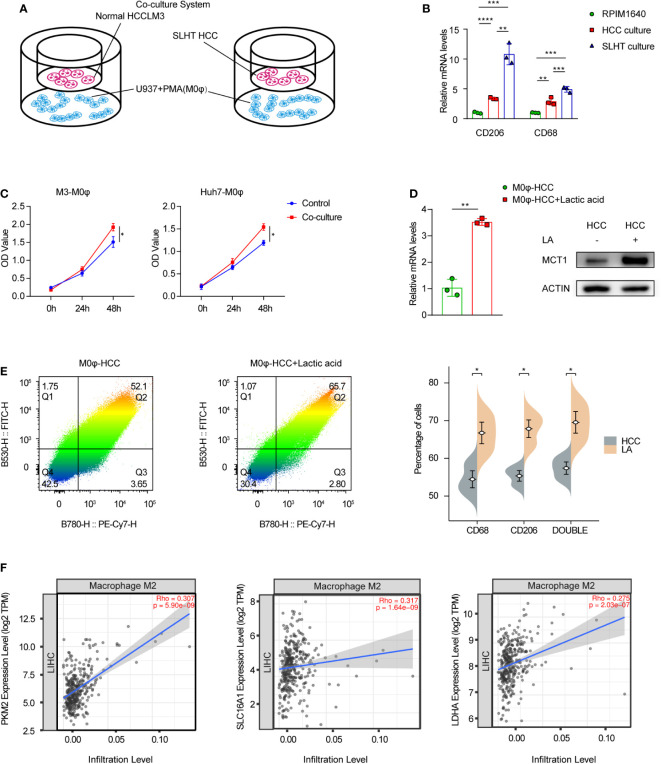
Accelerated M2 macrophage polarization in supernatants after sublethal lethal heat treatment. **(A)** Model of co-culture with HCC cells and U937 with PMA cells (M0φ). **(B)** qPCR of CD206 and CD68. **(C)** ELISA of lactic acid on normal co-culture medium or SLHT co-culture medium. **(D)** qPCR and western show the role of lactic acid on MCT1 during HCC co-culture with M0φ. **(E)** Flow cytometry shows changes in M2φ polarization markers. FITC: CD68; PE-Cy7: CD206. **(F)** the correlation of Macrophage M2 infiltration level and some lactic acid associated genes (PKM2, MCT1(SLC16A1) and LDHA). “NS”, not significant, *p < 0.05, **p < 0.01, ***p < 0.001, ****p < 0.0001.

M2-polarized macrophages have been documented to exhibit both anti-inflammatory properties and pro-tumorigenic activities. CD68 and CD206 serve as conventional markers for these cells. We established a co-culture model, with tumor cells in the upper layer and phorbol 12-myristate 13-acetate (PMA)-treated U937 cells (induced to M0φ phenotype) in the lower layer (**Figure 2A**). Our findings demonstrated a statistically significant elevation in CD68 and CD206 expression in the sublethal heat treatment group compared to the standard hepatocellular carcinoma group. Similarly, induced M0 macrophages exhibited upregulated mRNA levels of CD68 and CD206 (**Figure 2B**). An enzyme-linked immunosorbent assay (ELISA) of the supernatant further confirmed an increased lactic acid concentration in the co-cultured medium (**Figure 2C**). Consequently, co-culturing with SLHT HCC appears to expedite the polarization of M0 macrophages towards an M2 phenotype.

Considering the potential involvement of lactic acid in the polarization process post-sublethal heat treatment, we further analyzed MCT1 expression. Post 48-hour incubation with added lactic acid, both mRNA and protein levels of MCT1 were upregulated (**Figure 2D**), accompanied by an augmented proportion of M2 phenotype surface markers, CD68 and CD206 (**Figure 2E**). Upon consulting the TCGA database and analyzing the hepatocellular carcinoma dataset for immune cell infiltration, we observed that lactic acid-related genes, specifically PKM2, MCT1, and LDHA, all exhibited positive correlations with the infiltration level of M2-polarized macrophages, with statistically significant (**Figure 2F**).

### 3.3 MCT1 is a crucial molecule determining the promotion of M2φ polarization by SLHT and also inhibits M2φ pyroptosis

**Figure 3:**
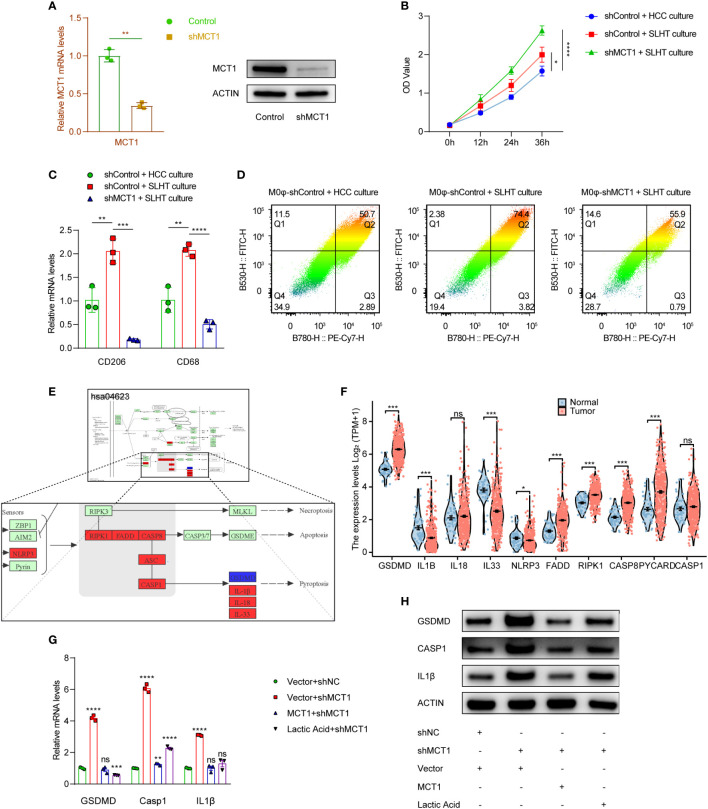
MCT1 is a crucial molecule determining the promotion of M2φ polarization by SLHT and also inhibits M2φ pyroptosis. **(A)** Establishing of MCT1 knockdown stable cells, and it was validated by qPCR and western. **(B)** ELISA of lactic acid on different conditions with shMCT1 and lactic acid. **(C)** qPCR showed the M2φ markers influenced by shMCT1 and lactic acid. **(D)** Flow cytometry showed the M2φ markers influenced by shMCT1 and lactic acid. **(E)** The change of some genes in HCC in KEGG pathway of pyroptosis referred by the infiltration of M2φ in TIMER 2.0 database. **(F)** The comparison of some genes in KEGG pathway of pyroptosis between HCC and para-tumor. **(G)** qPCR showed the changes of some pyroptotic genes influenced by shMCT1 and lactic acid. **(H)** western showed the changes of some pyroptotic genes influenced by shMCT1 and lactic acid. “NS”, not significant, *p < 0.05, **p < 0.01, ***p < 0.001, ****p < 0.0001.

We established U937 cells with a stable knockdown of MCT1 and induced their differentiation into M0 macrophages (M0φ) using PMA (**Figure 3A**). Preliminary investigation of the supernatants from the co-culture system with MCT1 knockout M0φ showed elevated lactic acid levels, suggesting that M0φ may have impaired lactic acid uptake in the absence of MCT1; whereas in the presence of SLHT HCC cells, the supernatants from the MCT1-knockdown group became higher in lactic acid OD value (**Figure 3B**). Notably, even in the presence of sublethal heat-treated hepatocellular carcinoma cells, there was a significant downregulation of M2 macrophage polarization markers in MCT1-depleted M0φ (**Figure 3C**). Further mass spectrometry analysis confirmed a marked decrease in M2 polarization markers in the absence of MCT1, underscoring the critical role of MCT1 in SLHT-mediated M2 macrophage polarization (**Figure 3D**).

Pyroptosis, a form of programmed cell death, plays a crucial role in cellular dynamics (12). Key molecules associated with the pyroptotic pathway (hsa04623) were identified using KEGG, and their expression was subsequently assessed in relation to M2 macrophage infiltration in hepatocellular carcinoma (HCC) using the TCGA LIHC database. Intriguingly, while many pyroptotic pathway molecules exhibited increased expression in HCC, the degree of M2 infiltration correlated positively with the expression of these genes (**Figure 3E, F**). A heightened expression of pyroptotic markers, such as GSDMD, CASPASE 1, and IL1β, was observed in MCT1-knockdown conditions, suggesting a potential rise in pyroptosis. However, the addition of exogenous MCT1 and lactic acid mitigated this increase (**Figure 3G, H**). Given that some studies report the inhibitory role of lactic acid in pyroptosis (13), it is plausible that lactic acid may, in part, enter M0φ via MCT1, suppress macrophage pyroptosis.

### 3.4 Lactic acid modulates macrophage phase separation, leading to a reduction in macrophage pyroptosis

**Figure 4:**
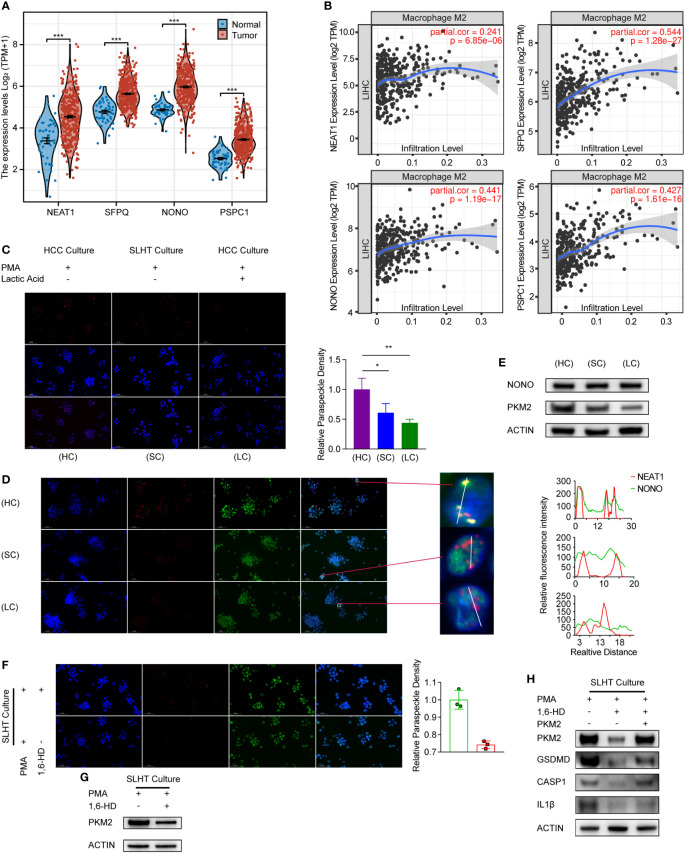
Lactic acid modulates macrophage phase separation, leading to a reduction in macrophage pyroptosis. **(A)** The comparison of 4 paraspeckle key genes (NEAT1, SFPQ, NONO, and PSPC1) in TCGA database between HCC and para-tumor. **(B)** The infiltration rate of Macrophage M2 in HCC base on the expression of 4 paraspeckle key genes (NEAT1, SFPQ, NONO, and PSPC1). **(C)** RNA scope of NEAT1 to present paraspeckle under the condition of SLHT and lactic acid. **(D)** RNA plus IP to present the change of NONO with the paraspeckle. **(E)** western showed the alteration of NONO and PKM2 under the condition of SLHT and lactic acid. **(F)** The effect of 1,6-HD on paraspeckle under SLHT culture. **(G)** The effect of 1,6-HD on PKM2. **(H)** western showed the alteration of pyroptotic markers influenced by 1,6-HD and PKM2. “NS”, not significant, *p < 0.05, **p < 0.01, ***p < 0.001, ****p < 0.0001.

At present, there is limited literature elucidating how lactic acid inhibits tumor-associated macrophages. Phase separation is a critical cellular biology process, with paraspeckles being a type of this phenomenon. We examined the TCGA database for key paraspeckle molecules in hepatocellular carcinoma (HCC) tissues compared to adjacent non-tumorous tissues. Our findings indicated an upregulated trend for these four crucial molecules in HCC tissues (**Figure 4A**). Additionally, upon reviewing the TIMER database, we observed that the infiltration level of M2φ positively correlates with the expression of these four molecules in HCC tissues (**Figure 4B**). This suggests a potential association between M2φ infiltration in HCC and paraspeckles. However, the relationship between paraspeckles and pyroptosis in macrophages remains unexplored.

NEAT1 serves as the structural backbone of paraspeckles. After staining for NEAT1, we found that the number of paraspeckles in the sublethal heat treatment group decreased by approximately 29.3% compared to the standard HCC co-culture group (*p* < 0.05). Intriguingly, after a 24-hour culture with additional lactic acid, the number of paraspeckles in macrophages further decreased by about 16.9% (p < 0.05) (**Figure 4C**). This implies a potential role of lactic acid in affecting paraspeckles within macrophages. We conducted a co-staining of NEAT1 and NONO under the same conditions (**Figure 4D**), revealing a decreased co-localization of NEAT1 with NONO, and a reduction in NONO speckles. This further emphasizes lactic acid’s role in diminishing paraspeckles in macrophages. Surprisingly, upon analyzing the key paraspeckle protein NONO, we found that neither sublethal heat treatment nor lactic acid induced changes in NONO’s protein level (**Figure 4E**). We hypothesize that phase separation variations may have led to spatial repositioning of NONO, rather than quantitative alterations in protein levels.

PKM2 is a pivotal molecule, with previous reports indicating its role in regulating pyroptosis (14). We noticed that its levels decrease during sublethal heat treatment, with lactic acid further diminishing its expression (**Figure 4E**). Thus, we speculate that phase separation may modulate PKM2. 1,6-HD, a known disruptor of phase separation condensates, has been used successfully to destabilize paraspeckle structures (15). After employing 1,6-HD, we observed a notable reduction in the number of paraspeckles (**Figure 4F**) and a significant downregulation of PKM2 protein expression (**Figure 4G**).

We further probed the effects of 1,6-HD on pyroptosis. 1,6-HD led to the downregulation of both PKM2 and molecules associated with the pyroptosis pathway. However, restoring PKM2 levels resulted in the re-elevation of GSDMD, Caspase-1, and IL1β (**Figure 4H**), suggesting PKM2 as a potential key mediator in paraspeckle-regulated pyroptosis.

## 4 Discussion

Our study integrates intricate cellular dynamics, metabolic shifts, and macrophage polarization, painting a comprehensive portrait of tumor-microenvironmental interactions under sublethal heat treatment. Our research indicates that sublethal heat treatment impacts the tumor microenvironment (TME) by promoting M2φ polarization through the reduction of paraspeckles.

The initial observations highlight a pH shift towards acidity in the tumor cell supernatant post sublethal heat treatment, which the study attributes to the heightened production of lactic acid. The significance of such acidic shifts in the tumor microenvironment is noteworthy, as acidosis can influence tumor progression, invasion, and resistance to therapy.

This research establishes lactic acid as a key player not only in inducing pH shifts but also in steering macrophage polarization. M2-polarized macrophages, with their anti-inflammatory and pro-tumorigenic functions, can remodel the tumor microenvironment, aiding tumor growth and progression. The elevation of lactic acid in the co-culture medium further strengthens its role as a polarization influencer. However, what drives tumor cells to increase lactic acid production upon sublethal heat treatment remains a question for further exploration.

The upregulated expression of MCT1 upon sublethal heat treatment is suggestive of its integral role in facilitating lactic acid transport, thereby contributing to the acidic microenvironment and M2 macrophage polarization. It would be interesting to further delve into the mechanism through which MCT1 modulates these shifts. The intriguing finding that there’s no significant difference in MCT1 expression between hepatocellular carcinoma patients and healthy liver tissues underscores the complexity of cancer biology and the multifaceted influences that drive tumor progression. Because the real tumor environment is more complicated than that of the tumor cell lines in the dish.

The tie-in with pyroptosis provides a fascinating perspective on how cellular death mechanisms intersect with macrophage dynamics in the tumor microenvironment. The relationship between lactic acid, MCT1, and the suppression of pyroptosis adds yet another layer of complexity. If lactic acid can indeed suppress macrophage pyroptosis, this could have broad implications for therapeutic strategies aimed at modulating tumor immune environments.

The observed link between paraspeckles (a type of phase separation) and macrophage dynamics in HCC tissues introduces another dimension to the study. The decline in paraspeckles post sublethal heat treatment and subsequent exposure to lactic acid indicates the multifaceted influence of lactic acid on cellular structures and functions. While the precise role of phase separation in tumor biology remains elusive, its implications in modulating cellular processes like pyroptosis require comprehensive exploration.

PKM2 stands out as a crucial molecule potentially bridging phase separation dynamics and pyroptotic pathways. The downregulation of PKM2 in conjunction with lactic acid exposure and sublethal heat treatment hints at its susceptibility to environmental cues. Using 1,6-HD, a phase separation disruptor, further cemented PKM2’s central role, especially when considering its potential as a therapeutic target.

We have mentioned some molecules in relation to immune cell infiltration in different Figures. There seem to be contradictions among them. For example, the lactate-associated molecule MCT1 promotes M2-like macrophage infiltration, but so does upregulation on paraspeckle-associated molecules; and upregulation of focal death-associated molecules also promotes M2-like macrophage infiltration. We believe that this only leads to the conclusion that these molecules may be related to M2-like macrophage polarization, a potential piece of evidence, but does not fully justify the conclusion. On the one hand, this is a comparison between cancer and paracancerous tissues from the TCGA-LIHC database, and not a comparison that occurs as a result of the changing conditions of the tumor-associated macrophages in this study; Furthermore, the conditions of the intervention are also the same as those in which metabolites due to cellular changes *in vitro* have a different effect on the tumor-associated macrophages. The infiltration of related molecules with M2- like macrophages in hepatocellular carcinoma cells needs to be explored in more studies.

This research underscores the intricate web of cellular dynamics, metabolic shifts, and external stressors in modulating tumor microenvironments. It establishes foundational links between sublethal heat treatment, lactic acid production, and macrophage polarization. However, it also raises several questions: We did not find the precise molecular mechanisms driving lactic acid upregulation upon heat stress; It is important to figure it out MCT1, PKM2, or the molecules involved in the pyroptosis pathway can be leveraged for therapeutic interventions in HCC or not.

Overall, this study paves the way for further research in tumor biology, particularly focusing on the roles of lactic acid, phase separation, and pyroptosis in cancer progression and potential therapeutic avenues.

## Data availability statement

The original contributions presented in the study are included in the article/supplementary material. Further inquiries can be directed to the corresponding authors.

## Ethics statement

Written informed consent was not obtained from the individual(s) for the publication of any potentially identifiable images or data included in this article because this is a public data on HPA database or TCGA database.

## Author contributions

ZF: Conceptualization, Data curation, Formal Analysis, Funding acquisition, Investigation, Methodology, Writing – original draft, Writing – review & editing. GY: Funding acquisition, Investigation, Methodology, Writing – original draft, Writing – review & editing. RKL: Investigation, Methodology, Software, Supervision, Writing – review & editing. XQ: Supervision, Validation, Writing – original draft. XY: Data curation, Formal Analysis, Methodology, Supervision, Visualization, Writing – review & editing. JW: Resources, Validation, Visualization, Writing – review & editing. ZY: Conceptualization, Investigation, Supervision, Visualization, Writing – review & editing. MS: Investigation, Methodology, Software, Supervision, Validation, Visualization, Writing – review & editing. WZ: Project administration, Resources, Software, Supervision, Validation, Visualization, Writing – review & editing. RL: Investigation, Methodology, Resources, Software, Supervision, Validation, Visualization, Writing – review & editing.
